# Supplementary material for: The Effect of Targeted Hyperoxemia on Brain Immunohistochemistry after Long-Term, Resuscitated Porcine Acute Subdural Hematoma and Hemorrhagic Shock
Source: Int J Mol Sci. 2024 Jun 14;25(12):6574. doi: 10.3390/ijms25126574 (PMC11204264; doi:10.3390/ijms25126574)
Supplement: Supplementary file 1 [file ijms-25-06574-s001.zip › ijms-2979300-supplementary.pdf]

| Parameter | Group | before ASDH & HS | after 2hrs ASDH & HS | 24hrs ICU care | 48hrs ICU care |
|---|---|---|---|---|---|
| MAP (mmHg) | Normoxemia | 98 (90;105) | 63 (61;72) | 119 (117;144) | 110 (101;155) |
| | Hyperoxemia | 99 (91;112) | 61 (57;67) | 131 (125;136) | 115 (109;124) |
| ICP ipsilateral (mmHg) | Normoxemia | 14 (11;16) | 16 (9;20) | 20 (14;24) | 39 (17;63) |
| | Hyperoxemia | 10 (9;16) | 9 (6;16) | 9 (1;17) | 15 (2;24) |
| ICP contralateral (mmHg) | Normoxemia | 12 (7;16) | 13 (7;22) | 15 (14;27) | 59 (15;62) |
| | Hyperoxemia | 9 (8;16) | 7 (4;16) | 8 (5;18) | 15 (9;24) |
| CPP ipsilateral (mmHg) | Normoxemia | 85 (76;91) | 52 (47;54) | 103 (101;120) | 89 (46;95) |
| | Hyperoxemia | 89 (79;107) | 52 (49;54) | 122 (119;124) | 99 (92;110) |
| CPP contralateral (mmHg) | Normoxemia | 88 (76;91) | 50 (42;56) | 106 (102;117) | 93 (42;95) |
| | Hyperoxemia | 91 (79;98) | 52 (49;59) | 122 (114;124) | 97 (93;107) |

**Table S1.** Mean arterial pressure (MAP), intracranial pressure (ICP) and cerebral perfusion pressure (CPP) before (Normoxemia: n=7; Hyperoxemia: n=7) and after (Normoxemia: n=7; Hyperoxemia: n=7) 2hrs of acute subdural hematoma (ASDH) and hemorrhagic shock (HS) as well as after 24hrs (Normoxemia: n=5; Hyperoxemia: n=6) and 48hrs (Normoxemia: n=3; Hyperoxemia: n=5) of ICU care.

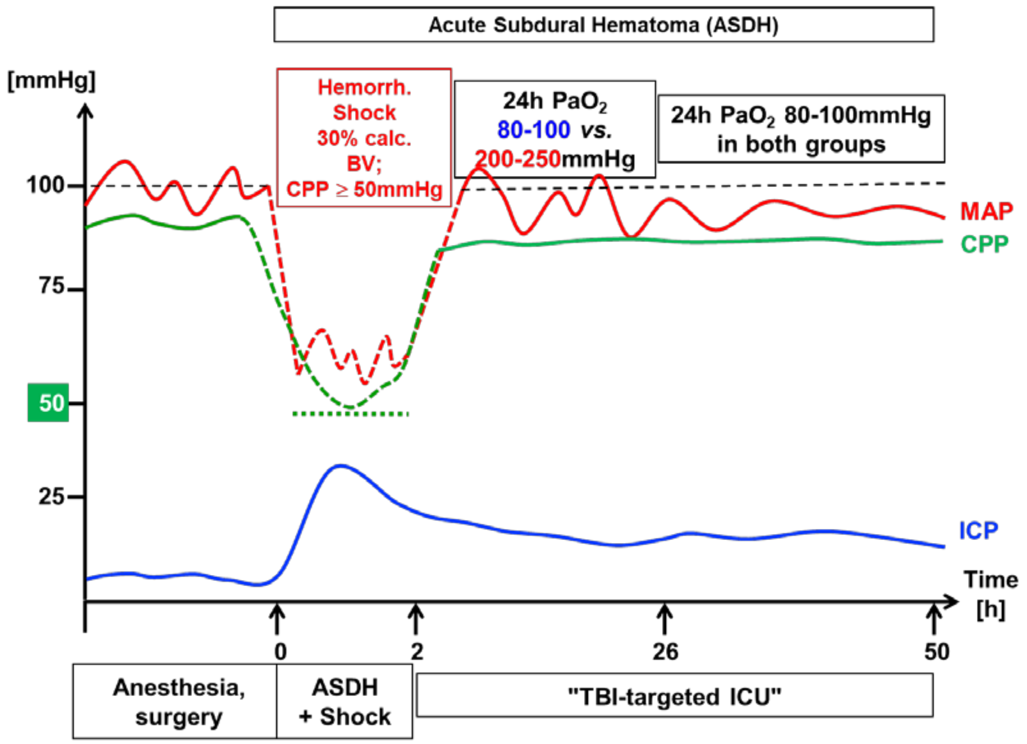


**Figure S1.** Experimental design. The dotted, dashed, or filled lines indicate schematic trends in intracranial pressure (ICP), cerebral perfusion pressure (CPP), and mean arterial pressure (MAP). BV: Blood volume.

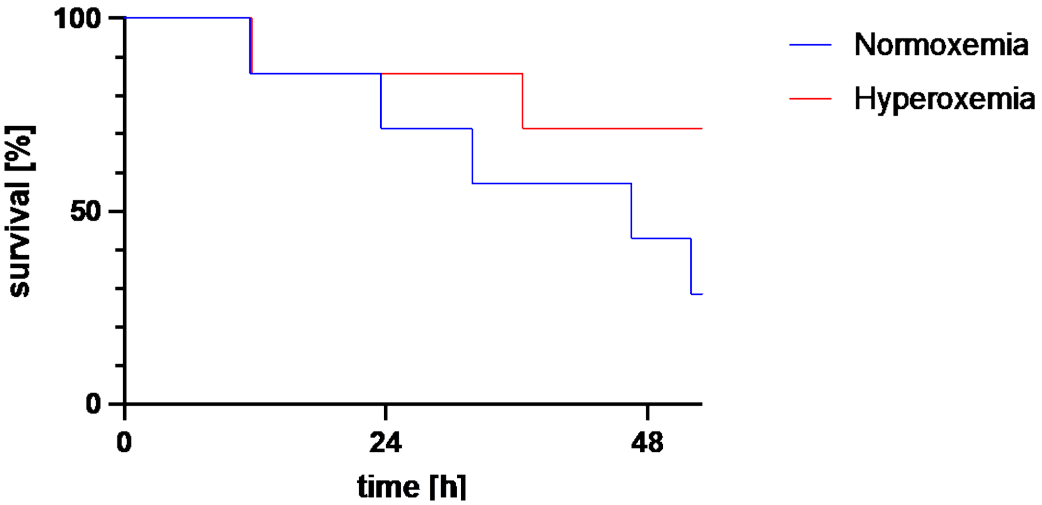


**Figure S2.** Survival analysis over a maximum of 53 hours of intensive care during resuscitation from acute subdural hematoma (ASDH) and hemorrhagic shock (HS) of animals treated with normoxemia (blue) or hyperoxemia (red), n=7.
